# Envisioning the Third Sector's Welfare Role: Critical Discourse Analysis of ‘Post-Devolution’ Public Policy in the UK 1998–2012

**DOI:** 10.1111/spol.12062

**Published:** 2014-01-24

**Authors:** Paul Chaney, Daniel Wincott

**Affiliations:** a Cardiff School of Social Sciences, Cardiff UniversityCardiff, UK; b Cardiff Law School, Cardiff UniversityCardiff, UK

**Keywords:** Third sector, Welfare pluralism, Policy, Discourse, Devolution, Framing

## Abstract

Welfare state theory has struggled to come to terms with the role of the third sector. It has often categorized welfare states in terms of the pattern of interplay between state social policies and the structure of the labour market. Moreover, it has frequently offered an exclusive focus on state policy – thereby failing to substantially recognize the role of the formally organized third sector. This study offers a corrective view. Against the backdrop of the international shift to multi-level governance, it analyses the policy discourse of third sector involvement in welfare governance following devolution in the UK. It reveals the changing and contrasting ways in which post-devolution territorial politics envisions the sector's role as a welfare provider. The mixed methods analysis compares policy framing and the structural narratives associated with the development of the third sector across the four constituent polities of the UK since 1998. The findings reveal how devolution has introduced a new spatial policy dynamic. Whilst there are elements of continuity between polities – such as the increasing salience of the third sector in welfare provision – policy narratives also provide evidence of the territorialization of third sector policy. From a methodological standpoint, this underlines the distinctive and complementary role discourse-based analysis can play in understanding contemporary patterns and processes shaping welfare governance.

## Introduction

Welfare state theory has often categorized states in terms of the pattern of interplay between social policies and the structure of the labour market (cf. Esping-Andersen 1990). Moreover, it has frequently offered an exclusive focus on state policy (Fraser 2009). In both cases, it has failed to fully recognize the role of the formally organized third sector. Added to this, such theory has given insufficient attention to the global trend of state restructuring and the rise of meso-governance. This study offers a corrective view. In 1998/99, government third sector policy in Scotland, Wales and Northern Ireland ceased to be decided by territorial ministries of central government. Instead, it is determined by administrations elected in three new political systems (re-)created by devolution (Chaney 2013b2013c). This has transformed the way party politics influence government policy on the third sector in the UK.

In the case of Scotland, the Scottish Labour Party and the Scottish Liberal Democrats formed successive executives until the 2007 elections; subsequently the Scottish National Party has held office. In Wales, the Welsh Labour Party has been in government since 1999 (including periods of coalition with the Welsh Liberal Democrats 2000–03 and Plaid Cymru 2007–11). In contrast, under the singular arrangements in Northern Ireland the exercise of executive functions has been done on the basis of power-sharing between parties. Accordingly, this article makes a distinctive contribution by focusing on policy discourse and how this transformation in territorial politics is impacting on the way that the third sector is envisioned as a welfare provider. It, therefore, addresses a key lacuna in contemporary understanding, namely how ‘welfare pluralism’ is shaped by the process of state decentralization.

In the following discussion, ‘welfare pluralism’ is a descriptive label which refers to the situation whereby service contributions by the voluntary and private sectors complement to state welfare delivery (Beresford and Croft 1983). The involvement of the third sector has long-standing links with political attempts to recast public service provision, yet emphasis on encouraging voluntarism and harnessing the contribution of the sector has heightened in recent decades (Hanlon *et al*. 2007). In definitional terms, we are mindful of Brenton's (1985: 57) rejoinder that ‘the voluntary sector's pluriformity and lack of clear boundaries do not lend themselves to the definitions and classifications upon which statistical methods are based’. In response, this article follows existing research practice (Casey 2004) by using the umbrella term ‘third sector’ to refer to the principal collective signifiers associated with non-government advocacy and service organizations; namely, ‘voluntarism’, ‘voluntary sector’, ‘third sector’, ‘civil society’ and ‘non-profit sector’.

The UK presents a propitious research context because the process of devolution initiated in 1998/99 has recast the territorial governance of the third sector with social policy responsibilities being transferred to newly (re-)established legislatures in Scotland, Wales and Northern Ireland. Such state restructuring can be seen as part of a wider ‘devolutionary trend [that] has swept the world [… involving widespread] transference of power, authority, and resources to subnational levels of government’ (Rodriguez-Pose and Gill 2003: 334).

In exploring the interplay between state restructuring and public policy discourse, we make an original methodological contribution to understanding the impact of devolution. Comparative discourse analysis is used to examine the key policy texts published by government in the constituent polities of the union state.^1^ The aim is to focus on the formative phase of policy-making and examine the contrasting political visions for the third sector as set out by devolved and central government through an emphasis on policy discourse. Accordingly, the article's principal aims are as follows:

1. To examine the framing of policy on the third sector, including its welfare role, in the four polities of the UK.
2. To examine the nature and development of social policy narratives related to the third sector in each territory.
3. To consider whether the data provide evidence of the territorialization of third sector policy in the wake of devolution.^2^

The remainder of this study is structured as follows. Following an outline of the research methodology the findings section consists of four parts:

1. *The policy framework prior to devolution*: analysis of policy framing in the state-third sector formal agreements or ‘Compacts’ of 1998.
2. *The policy framework following devolution*: exploration of the framing practices in the principal third sector policy documents in each territory 1999–2012.
3. Detailed examination of the visions of the third sector's welfare role in each polity (in the section ‘Welfare pluralism and the territorialization of third sector policy’).
4. Examination of the territorial policy narratives in each polity, reflection on their underlying causes and consideration of the application of our methodology to other liberal democratic regimes.

The main findings and their implications are considered in the concluding discussion.

## Methodology

The present method combines content and critical discourse analysis. In the former case, by recording the number of incidences of key words, ideas or meanings in a text, content analysis can further an understanding of the nature and salience of issues in policy formulation. In the current study, this is complemented by critical discourse analysis, operationalized here by frame analysis (Yanow 1999) of how, as key texts, policy documents enable policymakers to construct (or ‘frame’) measures to address social issues and effect change. The documents analyzed constitute the principal government policy documents on the third sector published in each of the four polities 1998–2012 (see ‘Policy Documents Included in Analysis’). Framing here refers to ‘collections of idea elements tied together by a unifying concept that serve to punctuate, elaborate, and motivate action on a given topic’ (Creed *et al*. 2002: 37). Our focus on salience and policy framing enables exploration of political narratives associated with the development of the third sector and its social policy role across polities.

This methodology was applied as follows. Electronic versions of each government's core policies on the third sector were analyzed using appropriate software.^3^ It should be noted that all of the texts analyzed were territorially discrete and referred to either: England, Scotland, Wales or Northern Ireland (as opposed to Great Britain or the UK). The policy texts were divided into ‘quasi-sentences’ (or an argument that represents the verbal expression of a single political idea or issue, Volkens 2001: 96) centred on the incidence of a key term.^4^ Thus each quasi-sentence was classified using an inductive coding frame based on key frames derived from the policy literature on the third sector (see ‘References’).

In order to offer a sophisticated exploration of policy discourse, this study adopted a tiered, or sequential, approach to the frame analysis in order to examine what Minsky (1975: 223) describes as ‘sub-frames’. These are explanatory or descriptive signifiers which attach to primary issue frames (in the case of the ‘welfare pluralism’ frame, they comprise the different underlying motives for cross-sectoral working – efficiency, effectiveness, choice, etc.). In terms of the temporal comparison in the analysis, the periods 1998–2003, 2003–07 and 2007–12 were used (each period is discrete, divided before and after the elections in a given year). This was a ‘best-fit’ approach aimed at capturing the first three terms of devolved government whilst acknowledging the fact that Westminster and meso-government operate on different electoral cycles.^5^ We apply the foregoing method in order to first compare policy framing in the state-third sector formal agreements or Compacts of 1998, and then to analyze the principal third sector policy documents in each territory, 1999–2012.

## Comparative Analysis of Policy Framing on the Third Sector 1998–2013

### The state-third sector compacts of 1998

**Figure 1:**
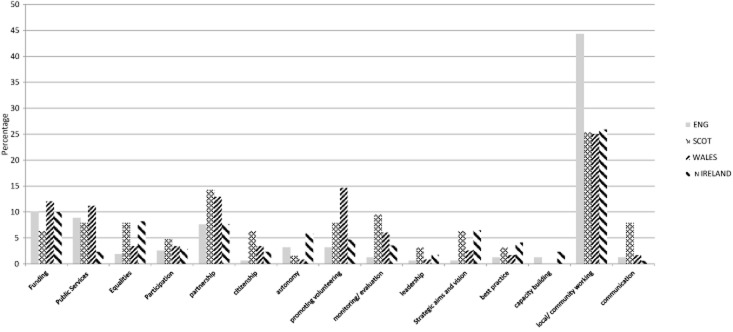
National comparison of policy framing in the state-third sector compacts (circa. 1998) *Note:* percentage breakdown by frame, each nation = 100%.

From an international perspective, the state-voluntary sector agreements or Compacts introduced in the UK in 1998 (see figure 1) were an innovation in contemporary governance; one that has subsequently been emulated in other countries. They are formal statements that set out mutual obligations and define each sector's role. As Kendall (2003: 2) observes, the ‘Compact idea is completely without precedent, representing an unparalleled step in the positioning of the third sector in public policy’. With a separate Compact for each UK nation they also prefigured devolution by adopting an explicitly territorial approach to state-third sector relations. A key question here is the extent to which there is continuity in the policy frameworks applying in each territory before and after devolution. In answer, critical discourse analysis reveals that whilst there are inter-polity differences in individual policy frames, overall there is broad consistency in the territorial framing profiles. The variance of the four data sets is not statistically significant (*P* = 0.432).^6^

The reason for this continuity lies in state-wide electoral politics. The Compacts have shared roots; each stems from New Labour's initial policy document, *Building the Future Together – Labour's Policies for Partnership between Government and the Voluntary Sector* (HM Government 1997). Thus, on the eve of devolved governance, notwithstanding some local variations, the framing in the four documents amounts to a general state-wide agenda on the values and priorities of state-third sector relations as set by a single party governing at Westminster. Thus, frames such as ‘partnership’ (where there is marked similarity in the discourse across territories) provide evidence of this shared framework. For example, in England reference is made to ‘working in partnership towards common aims and objectives. [… this] improves policy development and enhances the design and delivery of services and programmes’ (Home Office 1998: 8). At the same time the Scottish, Welsh and Northern Irish counterparts make similar assertions, ‘the government is concerned with promoting partnerships between public and voluntary sectors through its policies’ (Welsh Office 1998: 3); ‘the government will meet with the sector to develop policy and practice and promote effective dialogue’ (Scottish Office 1998: 10); and, the government will ‘involve the voluntary and community sector in partnership working and the process of developing and monitoring public policies’ (Northern Ireland Office 1998: 14).

However, the beginnings of distinctive territorial approaches to framing policy may also be detected in the Compacts. Examples include the disproportionately high level of attention paid to community and local level working in the English Compact (it accounts for 44.3 per cent of the overall policy discourse compared to a mean of 25.2 per cent for the other polities), typified by statements like, ‘it is important that the distinctive needs and interests of community groups are taken into account. A code of good practice will be developed to facilitate and reflect this’ (Home Office 1998: 12). A further example is the emphasis placed on third sector organizations' role in promoting equality in the Northern Ireland Compact; for example, ‘equality of access to resources and decision-making processes for all the people of Northern Ireland’ (Northern Ireland Office 1998: 11).

### Framing in the principal third sector policy documents in each territory, 1999–2012

An initial indication of the level of contrast or continuity in the framing practices pre- and post-devolution can be gained by examining the distribution of quasi-sentences made under each frame across the four polities 1999–2012. In other words, this is an aggregate measure of the total number of incidences of each frame in all key policy documents analyzed over the period broken down by territory. The result is empirical confirmation of the territorialization of policy. Statistically significant differences emerge in framing practices when the polities are compared in this way (*P* = <0.001) (see table1).^7^

**Table 1:** Inter-polity comparison of framing in third sector policy documents 1999–2012

Frame	Percentage of all frame references made in English policy documents	Devolved-polity mean (%)	N	χ^2^	*p*
Local/community partnerships	48.0	17.3	1143	1611.66	**
Autonomy	46.8	17.7	52	57.44	**
Capacity building	40.6	19.8	142	133.23	**
Funding	40.5	19.8	439	254.08	**
Partnership	40.3	19.9	247	138.77	**
Public services (non-specific)	40.2	19.9	459	293.58	**
Equalities	36.0	21.3	119	73.31	**
Citizenship	34.1	22.0	51	48.36	**
Best practice	31.2	22.9	91	65.37	**
Communication	27.1	24.3	47	21.08	**
Promoting volunteering	23.3	25.6	256	41.39	**
Monitoring/evaluation	23.0	25.7	197	131.92	**
Leadership	18.8	27.1	89	58.64	**
Community development	18.7	27.1	87	182.56	**
Participation	17.0	27.7	198	494.11	**
Strategic aims and vision	16.6	27.8	298	113.51	**

*Note:* ** = *p* < 0.001.

From a comparative perspective, the greater attention afforded to a number of frames in the English policy documents underlines how the ‘post-devolution’ policy discourse became territorialized. For a series of key frames the incidence of quasi-sentences is more than double the mean for the devolved polities. These include ‘local/community working’, ‘funding issues’ and ‘partnership’. Crucially, reflecting what our later analysis shows to be a greater emphasis on welfare pluralism in England (see below), the ‘public service delivery’ frame is given significantly more attention in the English public policy discourse than elsewhere (accounting for 38.1 per cent of all references under this frame, compared to 19.4, 11.4 and 31.1 in Scotland, Wales and Northern Ireland, respectively). However, territorial distinctiveness in framing practices is not restricted to England. It is also evident in the discourse relating to the devolved polities. Thus, policy in Northern Ireland accounted for most references to ‘capacity building’ (47.6 per cent), ‘strategic aims and vision’ (40.8 per cent) and, reflecting attempts to engage voluntary groups in civil conflict resolution (cf. Chaney 2012a), ‘leadership and political commitment’ (40.5 per cent). In contrast, Scottish policy predominated on ‘promoting volunteering’, a prominent trope in post-1999 debates (Fyfe *et al*. 2006) (32.9 per cent), as well as ‘communication’ (31.9 per cent) and ‘best practice/effectiveness’ (35.6 per cent). The Welsh policy framework placed particular emphasis on ‘participation’ (54.8 per cent of all references) and ‘citizenship’ (36.6 per cent); both are tropes in the inclusive governance discourse promoted by parties across the political spectrum to bolster initially fragile support for devolution (Chaney and Fevre 2001a).

### Welfare pluralism and the territorialization of third sector policy

A key welfare governance issue which attaches to state decentralization is whether there is policy continuity on third sector involvement in public service delivery spanning the pre-and post-devolution eras, or whether ‘devolved’ governance is fostering contrasting approaches in the constituent polities of the union state. In other words, we need to understand what happens to the way that the third sector's welfare role is envisioned when state-wide practices are replaced by four territorial political systems.

To explore this issue in depth, a two-tier methodology was employed. Following the initial coding process (see ‘Methodology’), all quasi-sentences associated with the public services frame in the key third sector policy documents covering the period 1999–2012 were (re-)coded according to sub-frames detailing the motive underpinning each reference to third sector involvement in service delivery (see table2). The results are striking and reveal statistically significant differences between polities (*P* = <0.001).^8^ This is significant because it confirms the rise of territorially distinctive approaches to welfare pluralism. Most notably, the policy framework covering the third sector in England stands out as the most ‘market-oriented’. In other words, it places greatest emphasis on the three elements defining this frame; namely, ‘securing better efficiency over state provision’, ‘added value’ and ‘marketization’ (*P* = <0.001).^9^ Just over a quarter (25.5 per cent) of references to third sector involvement in public service delivery in England related to the aforementioned motives, compared to 15.2 per cent in Scotland, and just 6.2 per cent and 5.9 per cent in Wales and Northern Ireland, respectively.

**Table 2:** Sub-frames: motives underpinning third sector involvement in service delivery 1999–2012

Sub-Frame	Scotland	Wales	England	N Ireland
Efficiency over state provision/added value/marketization	15.5	6.2	25.5	5.9
Community benefits/more responsive	17.4	21.4	10.5	16.8
Harness expertise/promote engagement/criticality	8.2	27.6	13.5	6.5
Greater effectiveness	17.9	8.2	10.2	10.0
Meet needs of disadvantaged	6.5	6.6	8.9	19.7
Increase service delivery capacity	9.9	2.9	8.8	8.2
Better coordination/complementarity	9.7	7.8	4.7	5.9
Social cohesion/good relations	3.9	3.3	2.5	16.5
Accountability	1.0	5.3	6.0	5.9
Better access/structural advantage/trust than state	1.7	7.4	2.4	2.4
Sustainability of services	5.8	0.4	2.6	1.8
User choice/personalisation of services	1.4	1.2	2.3	0.0
Ethos/values	1.2	1.6	1.8	0.6
N	414	243	1065	340

Further insight can be gained by examining of the development of substantive government policy on third sector welfare service delivery in England. This gained momentum in the wake of the UK government's 2002 and 2004 cross-cutting policy reviews (applying to England) (HM Government 2002, 2004). These set out the governing Labour Party leadership's aspiration for ‘a transformation of the third sector to rival the market and the state’ (Brown 2004). It was an agenda that was discursively packaged in a way that linked welfare pluralism to civic renewal (e.g. ‘voluntary and community sector organizations have a crucial role to play in the reform of public services and reinvigoration of civic life’, HM Government 2002: 2). Prefiguring current austerity measures, it also reflected pragmatism following the earlier round of cuts and down-sizing of the state sector in 1979–97. The discourse also included a frank admission that, as a result of diminished state capacity following the Thatcher and Major administrations, ‘we in government cannot do this on our own’ (HM Government 2002: 4). Rather, government's stated aim was ‘the building of strong civic communities; to reform the operation of public services and build a bridge between the needs of individuals living in those communities and the capacity of the state to improve their lives [… and to] take forward the development of social policy generally’ (HM Government 2002: 3).

Examples of the market-oriented discourse include, ‘encouraging the growth of a diverse and competitive market in which the third sector is expected to play a growing role’ (Cabinet Office 2006: 17); and ‘we believe in the power of competition to increase standards and deliver better value’ (HM Government 2012: 23). It is a discourse that spans the neo-liberalism of New Labour (1999–2010) and the subsequent Conservative-Liberal Democrat Coalition government (from 2010). Compared to the other polities, the English policy texts have a more assertive tone. This is particularly noticeable under the Coalition government, where it is often wrapped-up under its ‘localism’ agenda.^10^ For example, the self-stated aim of the latest iteration of the state-third sector Compact is:

Promoting contestability … these rights will give local community and third sector organisations the opportunity to challenge their local authority where they believe services or facilities would be better run by alternative providers. It will … give people a voice over local issues – also it will open up more contracts to third sector providers (HM Government 2010: 6).

A further notable aspect of the discourse is the way it is articulated in terms of the market discipline of competition, ‘government's public service reforms will enable charities, social enterprises, private companies and employee-owned co-operatives to compete to offer people high quality services’ (HM Government 2012: 3) and, ‘we will help the sector become more competitive in this emerging landscape, in particular through our new plans to run a series of commercial skills “masterclasses” in 2013’ (HM Government 2012: 5).

Compared to the other devolved polities, Scotland's greater embracing of the ‘market-oriented’ discourse is, in part, explained by the policy dynamic of the Labour Party simultaneously holding government office in Westminster and Edinburgh (cf. Laffin *et al*. 2007).^11^ Thus in the early-to-mid-2000s, the Scottish Executive followed key aspects of Westminster policy (Hassan and Shaw 2012). This is evident in the policy texts. For example, ‘the UK review [covering England] identified several obstacles and challenges that need to be overcome to enable the sector to develop its public service delivery role. These included the following development needs *which apply equally within a Scottish context*’ (Scottish Executive 2004: 8, emphasis added). Examples of the Scottish market-oriented discourse include, ‘the social economy in Scotland is becoming much more business-like in its approach to service delivery – and this is helping some organisations to generate significant profits on some of their services. Some might call them not-for-profit businesses. But we see them as more-than-profit organisations’ (Scottish Executive 2004: 5).^12^ Here the stated aim was ‘to make Scotland a world leader in the development of an enterprising third sector … [to] develop new services and [for them to] market themselves effectively’.

A further common trope crosscutting the market-oriented discourse in the English and Scottish policy frameworks is the pervasive – yet empirically contested – notion of non-state organisations' superior ability to innovate in service delivery (cf. Borins 2001). For example, ‘the best organisations [have] … an ability to be flexible, offer joined-up service delivery and … the experience to innovate’ (HM Treasury and Cabinet Office 2007: 49) and, ‘we recognise the added value that the sector brings to the delivery of public services. Social economy organisations have a real understanding of the area in which they operate. They are flexible and able to innovate’ (Scottish Executive 2005a: 2).

In Wales, at a rhetorical level at least, Labour has been keen to espouse the existence of putative ‘clear red water’^13^ between itself and the Party at Westminster. Instead, it has preferred to style itself as ‘Classic’ rather than ‘New’ Labour (Chaney and Drakeford 2004), thereby signalling its opposition to the latter's neo-liberal agenda. Such a standpoint is evidenced by the Welsh party affording less attention than its English counterpart to the market-oriented sub-frame. Nevertheless, analysis shows welfare pluralism is still a feature of the third sector policy framework in Wales. Examples include, ‘the model, which we have opted for, seeks to maximise efficiency gains through the scale economies of more effective co-operation and coordination between agencies across the whole of the public sector, not excluding the independent, voluntary and private sectors’ (WAG 2004: 3) and, there must be ‘a willingness to consider new ways of providing services, including an increasingly mixed economy of provision, with the potential for a greater role for the third sector in delivery’ (WAG 2006: 21).

Compared to the English policy documents, the present analysis reveals that the policy frameworks in the devolved nations place greater emphasis on extolling the community benefits of third sector involvement in welfare service delivery. According to the devolved administrations' policy discourse, third sector involvement is generally more responsive than services provided by the state. Thus the ‘community benefits’ of service delivery by third sector organizations, or welfare pluralism, is a sub-frame that constitutes 17.4 per cent, 21.4 per cent and 16.8 per cent of all references in Scotland, Wales and Northern Ireland, respectively – compared to 10.5 per cent in England. It is typified by the assertion by the Northern Ireland Executive that ‘effective partnership between Government and the Voluntary and Community Sector can make a valuable contribution to more responsive and people-centred public services’ (DSD 2011: 2).

The idea that third sector organizations deliver more effective services than the state receives greatest attention in the Scottish policy discourse (it accounts for 17.8 per cent of references, double the mean for the other polities). Examples include, ‘cross-boundary solutions … are increasingly being used by local authorities to generate efficiencies and to ensure more effective delivery of services’ (COSLA/SCVO 2009: 5) and, ‘recent years have seen increasing numbers of voluntary sector organizations playing an effective part in delivering on key agendas in terms of service delivery’ (Scottish Executive 2004: 6).

In contrast, and reflecting the more comprehensive equalities law applying in the province (Chaney 2012a), the involvement of third sector organizations in service delivery in order to meet the needs of disadvantaged groups receives three times the level of attention in Northern Ireland compared to the other polities (19.7 per cent of references compared to a mean of 7.3 per cent in the other territories). Examples include, ‘voluntary and community organisations have a track record of tackling social need and deprivation and are well placed to develop and deliver improved frontline services, particularly to the most disadvantaged people in society’ (DSD 2005a: 18; see also DSD 2005b).

Lastly, the analysis shows the policy discourse in Wales to have given greatest attention to the idea that third sector organizations are subject to higher levels of trust than state service providers (see Taylor 2002) (7.4 per cent of references compared to a mean of 2.1 per cent elsewhere). For example, ‘we believe the third sector is in a particularly strong position to provide frontline services when users have multiple disadvantages … the service is targeted at users who are likely to mistrust businesses or state providers’ (WAG 2006: 27; see also WAG 2009).

Before summarizing the implications of the present findings we first consider the territorial policy narratives in each polity, and reflect upon the apparent drivers as well as the transferability of this study's methodology to other liberal democratic regimes.

### Territorial policy narratives

The following examination of policy framing alongside the principal policy developments in each polity (see table3) reveals the structural narrative of the third sector in each territory. It is a technique that offers a temporal perspective of how frames as narrative devices, develop and become more or less prominent and persuasive over time (Petersen and McCabe 1983). In this way it enables an ‘understanding of the dynamics and frameworks of decision makers that supports and articulates policy choices and the claims underlying them’ (Roe 2011: 541).

**Table 3:** Territorial narratives: key themes and tropes in third sector policy frameworks 1999–2012

	Scotland	Wales	England	Northern Ireland
1998–2003	Raising Awareness, improved monitoring arrangements;^1^ ‘ensure that, in the process of policy-making … volunteering and community groups are considered and taken fully into account.’^2^	‘Encourage co-operative methods of decision making; review performance, encourage volunteering initiatives … will maintain … a policy on working in partnership with the voluntary sector and measures to support this’.^3^	‘Community groups can play a range of different roles, including … build[ing] social capital and community cohesion; and delivering services, often locally and informally, based on their assessment of community needs’.^4^	‘work together as social partners to build participative, peaceful, equitable, and inclusive communities … open up opportunities for more active participation by the voluntary and community sector in developing public policy’.^5^
2003–2007	‘to embed a robust culture of volunteering in Scotland’;^6^ develop the third sector: ‘as a service delivery partner, in building strong communities, in advocacy and developing policy thinking, As an agent of change’.^7^	Promote: ‘active citizenship; measure and recognise what matters to the voluntary sector; and, assess carefully, in consultation with relevant voluntary organisations, the potential impact of policy changes upon the sector’.^8^	‘Transforming public services: engaging users, empowering communities … the Government is committed to ensuring that, where a diverse range of providers is being developed, we positively encourage the involvement of third sector organisations in the design and delivery of public services’.^9^	‘A key role in … human rights, equality and good relations; build better relationships within and between communities to tackle sectarianism’.^10^
2007–2012	Strategic commissioning and procurement, ‘deliver shared services … deliver Best Value and maximised capacity within the sector’;^11^ opening markets to an enterprising third sector, promoting social entrepreneurship; providing support for business growth; raising the profile of enterprise in the third sector'.^12^	‘Empowering people and communities; Valuing voluntary action; Strengthening communities; Strengthening public/third sector engagement; Enabling raised performance and growth’.^13^ ‘Equal opportunities for service users to use the Welsh and English languages in the Third Sector …’.^14^	‘Delivering innovative and personalised public services’.^15^ ‘Helping CSOs to challenge existing provision of services, access new markets and hold government to account’.^16^ ‘Empowering communities … Opening up public services … Promoting social action.’^17^	‘Contribute to a more cohesive civil society. Effective partnership; people centred public services: strengthening communities and harnessing expertise in helping design better public policy and services’.^18^

*Sources*: 1 = Scottish Executive 2001; 2 = Scottish Executive 2003a; 3 = NAfW 2001; 4 = Cabinet Office 2003; 5 = NIO 1998; 6 = Scottish Executive 2006; 7 = Scottish Executive 2005a; 8 = NAfW 2004; 9 = Cabinet Office 2006; 10 = DSD 2005a; 11 = COSLA/SCVO 2009; 12 = Scottish Government 2008; 13 = WAG 2008; 14 = WAG 2010; 15 = Cabinet Office 2009; 16 = HM Government 2010; 17 = HM Government 2010; 18 = DSD 2011.

#### Scotland

A key aspect of the narrative in Scotland is far-reaching structural change affecting the third sector (e.g. Scottish Executive 2003b, 2005b). During the early years of devolution the Scottish Executive supported a tripartite system of Volunteer Centres, Councils for Voluntary Service and Social Enterprise networks (for a discussion, see Fyfe *et al*. 2006). Following its election in 2007, the Scottish National Party government introduced a Concordat between Scottish government and local authorities; a development which has recast state-third sector relations. Its effect has been to devolve power and give local authorities greater control in dealings with the sector. On one level this is nothing new. It is part of the wider international trend of promoting localism evident over recent decades (cf. Page 1991). Yet the means by which it has been pursued in Scotland are distinctive. Since 2011, new third sector ‘interfaces’ have been developed in each community planning area. The aim is to align the sector with the Community Planning Partnerships and Single Outcome Agreements. In turn, this is designed to support to local third sector organizations, boost volunteering and develop social enterprise. Crucially, its proponents claim that it allows a more strategic approach to the third sector's welfare role.

Framing data provide further details of the developing policy narrative (see figure 2a). Over the period 1999–2012, the greatest increase has been in the ‘promoting volunteering’ frame (+23.7 percentage points); for example, the ‘Scottish Executive will … ensure that volunteers are supported and encouraged in every possible way’ (Scottish Executive 2004: 2). The underlying concern here is to increase the capacity of the sector to deliver services as well as to boost active citizenship. Recent work (Asenova *et al*. 2012: 17) reveals how, in the face of growing austerity, local authorities' principal strategic policy response is ‘service transfer’ to the third sector. Such a trend is supported by the framing data which show an increase in proposals under the ‘public services’ frame (+14.7 percentage points) underlining the growing emphasis on welfare pluralism as a response to the current recession and cuts in welfare spending (estimated by the Scottish government to be £4.5 billion in the five years to 2014–15).^14^ In contrast, the frames ‘monitoring and evaluation’ (−11.5 percentage points), ‘communication’ (−7.2 percentage points) and ‘best practice’ (−5.9 percentage points) have all declined in salience since 1999.

**Figure 2a:**
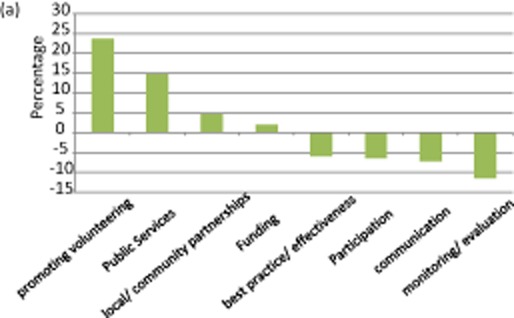
Key shifts in policy framing: Scotland, 1999–2012

#### Wales

A core aim at the outset of constitutional reform in Wales was ‘establishing a new, more inclusive and participative democracy’ based on devolved government ‘working in partnership with the voluntary sector’ (Welsh Office 1998: 3). Uniquely, this commitment was enshrined in the subsequent devolution Acts such that statute requires Ministers publish a Scheme setting out how they propose to engage with and promote the interests of voluntary organizations (Chaney and Fevre 2001b2001a). Accordingly, successive Welsh administrations have sought to develop third sector capacity and mainstream the sector into the conduct of public business (see figure 2b).

**Figure 2b:**
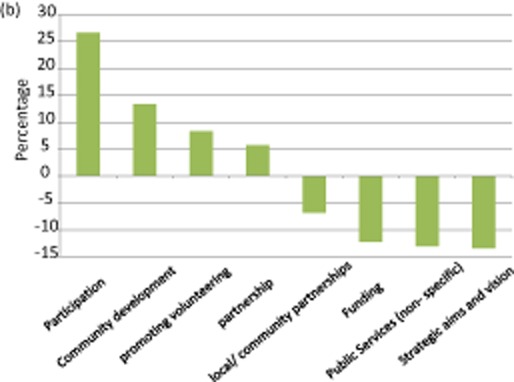
Key shifts in policy framing: Wales, 1999–2012

As a result, the policy narrative in Wales is one of increasing attention to ‘participation and engagement’ (+26.6 percentage points), ‘community development’ (+13.3 percentage points) and ‘promoting volunteering’ (+8.3 percentage points [Chaney 2013a2013c]). Examples of this discourse include, ‘we are also keen to promote greater partnership working between third sector organisations themselves, not only to ensure a stronger voice for citizens locally, but also to improve efficiency through the sharing and pooling of capacity’ (WAG 2008: 47). A seemingly counterintuitive finding is the decline in the salience of ‘funding issues’ (−12.2 percentage points) and ‘third sector involvement in public service delivery’ (−13.0 percentage points). This is a key difference compared to Scotland. There are two main explanations. As noted, successive Labour Party-led administrations in Wales have been keen portray themselves as champions of state provision of social welfare; thereby distinguishing themselves from the mixed economy approaches espoused in England and Scotland. In addition, the most recent key policy document in the Welsh dataset is from 2010 (as opposed to 2012 in the Scottish case). At that juncture, the full extent of government austerity measures was not appreciated. The latest data reveal the full gravity of the situation and point to the likelihood of revised framing practices in future rounds of policy-making and an attendant shift towards welfare pluralism. Underpinning this scenario is a reduction of benefit and tax credit entitlements in Wales of £590 million a year by 2014–15 and, as Crawford, Joyce and Phillips (2012: 12) state, ‘real-terms reduction in [Welsh government] current spending of 8.4 per cent between 2010–11 and 2014–15, with the capital budget falling by 42.8 per cent’ (see figure 2c).

**Figure 2c:**
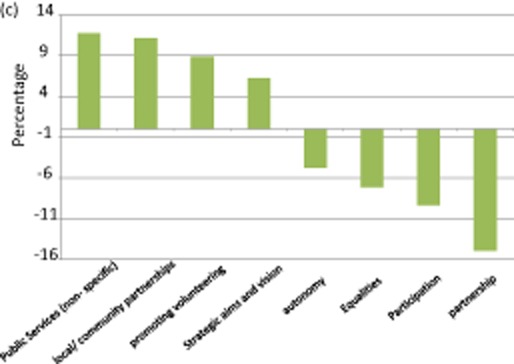
Key shifts in policy framing: Northern Ireland, 1999–2012

#### Northern Ireland

In Northern Ireland, the structural narrative of the third sector forms part of the wider project to secure inclusive governance in the wake of the civil conflict (Chaney and Rees 2004; DSD 2007a, 2007b, 2008, 2012). Thus the Compact agreed by the government and the sector in 1998 (NIO 1998) espoused the themes of accountability, active citizenship and the participation of the sector in the development of public policy. It is an agenda facilitated by the Joint Government – Voluntary and Community Sector Forum which was established to promote discussion of general issues of common concern. The re-imposition of direct rule and successive cross-government policy reviews (DSD 2006, 2011; PWC 2004) underline the many challenges facing the sector. Notably, government's second strategy (DSD 2006) prioritized ‘building communities’ and ‘targeting disadvantage’. In 2008 (and following a period of direct rule), a resolution of the Assembly called on the Executive to set out further measures to strengthen co-working and engagement with the sector.^15^ After protracted negotiations a successor to the Compact was published in 2011 (DSD 2011).

This singular policy history has resulted in a post-1998 structural narrative characterized by an increase in policy framed in terms of ‘local/community co-working’ (+19.6 percentage point) and, significantly, third sector involvement in public service provision (+10.8 percentage points). For example ‘we will encourage and support more effective and wider-ranging involvement of voluntary and community organisations in the delivery of public services’ (DSD 2005a: 4). As in the case of Scotland, the growing emphasis on welfare pluralism is driven by austerity measures. This is evident in the province's Budget settlement 2011–15 which sets out ‘a real terms reduction in resource DEL [Departmental Expenditure Limit] of 8 per cent, and a real terms reduction in capital DEL of 40 per cent by the end of the Spending Review period’ (NIE 2011: 21). The role of the recession in promoting service transfer from state to third sector is underlined by the fact that over a half (55.6 per cent) of post-1998 references to service delivery have been made since 2007 (see figure 2d).

**Figure 2d:**
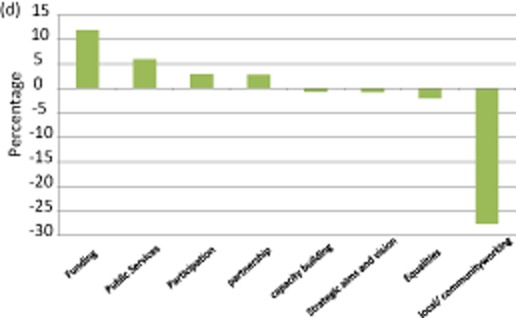
Key shifts in policy framing: England, 1999–2012

#### England

The structural narrative in England is characterized by a strong post-1998 emphasis on welfare pluralism (Home Office 2003, 2004, 2005). In contrast to the other polities – and reflecting the neo-liberalism of the Blair administrations – the mixed economy discourse is evident from the outset and is a core feature of the three successive Compacts between the sector and government (cf. Cabinet Office 1998a, 1998b, 1998c, 1998d, 2009; HM Government 2010). It has been subject to renewed emphasis by the current Coalition government such that policy is concerned to make it ‘easier for civil society to work with the state’. For example, ‘we are making it easier for civil society to … access public service contracts … help the sector become more competitive … [and] identify opportunities’ (HM Government 2010: 4). Once again reflecting the role of austerity in driving a mixed economy of welfare approach, almost two-thirds (63.7 per cent) of the post-1998 total of references to funding and 39.5 per cent references to public services have been made since 2007.

#### Summary

The foregoing territorial narratives highlight the power dimension to the third sector's changing welfare role. This, and the discursive processes which drive the policy process in territorial political systems with multi-party elections, are not unique to the UK. Thus the present methodology is applicable to other liberal democratic regimes where it may complement existing structural analyses. Examples of the power dimension to the envisioning of the sector's welfare role in the present study include national administrations' framing of third sector policy in terms of autonomy, independence and localism. Yet, as the foregoing analysis suggests, the underlying power dynamic is one of central authorities *retaining* power in the allocation of resources as well as ‘high level’ policy and law-making functions, whilst transferring to local government and third sector bodies day-to-day service delivery responsibility along with the attendant political risks (policy examples include the Scottish Community Planning Partnerships and the Localism Act 2011 in England).

The power dimension is also evident in the discourse on welfare pluralism. Notably, the foregoing analysis reveals this to be framed in terms of empowerment of the sector and the communities that they serve. Yet, here austerity is a key under-acknowledged driver; one that spans polities. In this regard, the third sector provides the political elite with a viable means to deliver welfare outside of state provision whilst avoiding exclusive reliance on the private sector. Again, the power dynamic is one of government retaining control but transferring political risk in the form of delivery responsibility (as well as answerability to regulators and budgetary oversight).

In an era of multi-level governance, the present comparative structural narrative methodology also reveals how welfare pluralism is both a ‘devolved’ and shared construct. In other words, economic imperative underpins its commonality across polities, yet distinctive framing practices apply in each (in turn, reflecting territorial party politics). This means that it is advanced and applied in different ways (e.g. as the contradictory elements in the Welsh public policy discourse attest – at once underlining the need to maintain and support state provision yet embracing non-state provision).

The structural narrative approach also underlines the way in which policy change is presented in order to appeal to, and meet the expectations of, specific audiences. This is captured in the literature on strategic framing (cf. Pan and Kosicki 2007) whereby common policy objectives are advanced by use of contrasting policy frames. In the present case, it is again typified by the discourse on welfare pluralism. Thus compared to the Welsh and Northern Irish – and to a lesser extent the Scottish policy discourses (which all underline co-working, partnership, local engagement, and are pitched to the support base of the Left-of-centre parties which have predominated in devolved government) – the neo-liberal UK coalition government covering England places greater emphasis upon efficiency, competition, accountability and innovation in relation to non-state provision of welfare (tropes that ‘play well’ with traditional Conservative Party supporters).

## Discussion

By focusing on the changing ways in which territorial politics envisions the third sector's role as a provider of welfare, the present study complements a vast body of macro-comparative studies of welfare regimes (classically, Esping-Andersen 1990). Yet whilst this literature has placed increasing, if inconsistent, attention on the place of voluntary associations and informal care as pillars of welfare governance alongside the state and (labour) market (Arts and Gelissen 2010), it has given insufficient attention to the welfare role of the formally constituted third sector. Moreover, it has largely ignored the way that shifting sectoral welfare roles play out in the context of devolution and the global rise of multi-level governance. The evidence from the UK shows these to be significant factors shaping the nature of social welfare in the 21st century.

In methodological terms this study marks an initial step in investigating the nexus between policy discourse, state restructuring and welfare. However, the limitations of the findings also need to be acknowledged in that they are derived from examination of a specific type of policy discourse; albeit the principal statements of government policy in each territory. To this end it is suggested that future study needs to explore the policy discourse and deliberations of exogenous interests, most notably civil society organizations and policy networks, as they set out their vision for the sector. It also needs to examine the role of state-wide versus regionalist parties in shaping the nature and extent of policy convergence/divergence across territories – as well as the extent to which civic nationalist parties' influence on third sector policy is shaped by the twin imperatives of (sub-)national autonomy and state-building. In addition, this study's focus on executive policy documents needs to be complemented with analysis of legislative proceedings, including backbenchers' and opposition parties' discourse as they seek to (re-)define the sector's welfare role.

Earlier work shows that, in policy terms, the impact of devolution on the third sector in the UK was not immediately apparent. Thus, one study of the early 2000s concluded that, ‘although there is evidence of significant structural change in the forums for engagement flowing from devolution, there has been less to suggest significant policy divergence’ (Alcock 2009: 9). In contrast, we have been concerned to explore the new *formative processes* which now shape third sector policy. This has explanatory power to complement traditional analysis of policy outputs, and, as the current discourse data show, reveal the ongoing territorialization of social policy following the redrawing of political boundaries in the union state.

The latter is a function of the fact that the interlocutors in the four post- 1998/99 state policy-making systems in Scotland, Wales, Northern Ireland and England are markedly different in number, political complexion and influence to those found at Westminster before 1998. Crucially, ‘post-devolution’ policy-making is grounded in starkly contrasting notions of identity, culture, as well as political and constitutional ambitions for the nature and functioning of the modern state (Chaney 2013c). The present methodology reveals this, yet it also underlines that the changes affecting the third sector in the UK are more than just structural in nature; they are also about the situated internal party politics of state-wide parties as they seek to manage tensions between their UK role and presence in the devolved nations. This was notable, for example, in the discourse on the role of the private sector and market-based approaches to welfare (with strong continuity between Scottish Labour and ‘New’ Labour at Westminster, yet clear contrasts with Welsh Labour). Accordingly, this study's process-oriented approach underlines the complex and contingent ways in which the territorial politics of devolution shapes the third sector's welfare role.

The rise of ‘sub-state’ welfare pluralism also needs to be viewed within the context of the third sector's importance in the current economic climate. The latter is a product of the development of state-third sector relations over the past two centuries. A period which spans ‘the development of the “new philanthropy” in the late 19th and early 20th centuries [and …] the expansion of state welfare provision after 1945’ (Harris 2010: 25; Wincott 2011, 2012). Over the past century, governments from both Right and Left have advocated co-working with the sector, albeit in contrasting ways and with varying motives. The post-2008 global recession has added fresh impetus to this agenda. As Wilding (2010: 97) cogently observes, it ‘has shaken the confidence of and prospects for the UK voluntary and community sector … Looking forward, managing relationships with government in a period of substantive public expenditure cuts is likely to be the biggest test of the sector's ability to survive and even thrive, in a recession’. Indeed, a growing body of empirical work reveals the impact of austerity and the ways in which financial constraints are limiting organizational effectiveness. According to Brown *et al*. (2013: 58), this underlines ‘a contradiction between the current [UK] coalition government's “Big Society” ideas^16^ and the reality as it unfolds in … the third sector’. The present, therefore, marks a critical juncture:

if ever there was momentum to roll back the welfare state, it is the (aftermath) of the financial crisis of 2008–09. All theoretical perspectives within comparative welfare state research predict radical reform in this circumstance … budgetary constraints are forcing political actors to make tough choices and introduce austerity policies. As a result, the question of who pays what, when, and how will likely give rise to increasingly sharp distributional conflicts (Vis *et al*. 2011: 338)

Secondary data underline this point and explain why parties from across the political spectrum view the third sector as an appealing option to make good any shortfall in welfare provision arising from public sector cuts (notwithstanding sharp divisions on how this should be operationalized). They reveal that the efforts of the 10.6 million people in the UK who volunteer once a month contributes the equivalent to the work of 1.3 million full-time employees at a potential cost to the state of £23.1/US$35.9 billion (based on median hourly wage) (NCVO 2011: 21).

Thus the present study underlines how the contrasting policy frameworks in the four UK polities continue to be tempered by the recession. Prior to 2007/08, the devolved governments' policy discourse on mixed economy approaches to welfare was often driven by more expansive visions of welfare (compared to Westminster). Post-recession, they are increasingly shaped by the administrations' differing responses to austerity, thereby giving added impetus to policy divergence. This economic dimension to welfare pluralism is certain to accelerate in future years owing to:

1. the greater size (compared to England) of the public sector(s) in the devolved nations (with inherent vulnerability to downsizing as part of ongoing austerity measures); and
2. the recent devolution of significant taxation and borrowing powers to Scotland (with Wales likely to follow).

The latter transition to a quasi-federal taxation system post-dates the current dataset. Yet it will mean that meso-governments' embracing of third sector welfare delivery will no longer be informed by the general necessities of block grant transfers from the UK Treasury. Instead, it will be shaped by a new economic imperative as devolved administrations take on the political risks associated with raising direct taxation, determining the level of welfare spending^17^ and, crucially, its allocation between sectors.

Overall, the foregoing analysis reveals the key significance of devolution to the third sector. The new territorial politics associated with the creation of four distinct political systems in the UK means that policy is now shaped by the contrasting ways that parties in the constituent polities envision the sector's role. This is (re-)defining the nature of contemporary welfare and driving third sector policy divergence within the union state. Sub-state electoral politics and ongoing constitutional change mean that, in the new millennium, the direction of travel appears to be firmly away from predominantly state-wide policy-making which characterized social welfare for much of the 20th century.

## Footnotes

1 ‘Union state’ refers to the United Kingdom – a state founded on the union of England and Wales, followed by unions with Scotland and Ireland (subsequently, post-1920, Northern Ireland).

2 The territorialization of policy is a spatial process whereby, compared to past practices, policy decisions are made at a more local level and tailored to local circumstances (see e.g. Chaney 2012b).

3 Adobe Reader XI.

4 For example, the single statement, ‘our approach will amend the schools curriculum to encourage young people to undertake voluntary work in order to help others and benefit the area in which they live’ consists of two quasi-sentences: (a) ‘encourage young people to undertake voluntary work’, a quasi-sentence coded under the ‘volunteering’ frame; and (b) ‘benefit the areas in which they live’, a quasi-sentence coded under the ‘community development frame’.

5 It is an inexact match because devolution in Northern Ireland pre-dated devolution elsewhere by a year (and has been characterized by the re-imposition of periods of direct rule). Moreover, 2012 was added to the last period in order to capture the latest policy documents.

6 F-test, df = 3, F = 0.93.

7 χ^2^ = 2704.213, df = 27, P = <0.001; F-test df = 1, F = 6.54, P = 0.015489.

8 χ^2^ = 362.573, df = 27, P = <0.001.

9 χ^2^ = 477.571, df = 3, P = <0.001.

10 An agenda axiomatically to devolve decision-making to local communities, given legislative effect in the Localism Act 2011.

11 The Scottish case is explained by internal Labour politics and a close alignment between the Edinburgh and Westminster leaderships (see Laffin *et al*. 2007). This was initially true of Wales, yet superseded by a political determination to be seen as different to New Labour following the departure of Alun Michael AM as Welsh First Minister (see Flynn 1999).

12 It should be noted that this policy document seems to be using a rather unusual definition of what is in the ‘third sector’ and also that this very confusion in the text is itself an important finding from the data. It reflects the policy standpoint which says that what is being sought is any alternative to state/governmental service provision and there is no particular preference for the third sector.

13 ‘Clear red water’ is the term used by the then Welsh First Minster, Rhodri Morgan AM, in a keynote speech in 2002 to signal the difference in approach to welfare between the Welsh Labour Party and Tony Blair's New Labour Party in England. The speech is available at http://www.sochealth.co.uk/the-socialist-health-association/sha-country-and-branch-organisation/sha-wales/clear-red-water/ (accessed 31 October 2013).

14 http://www.scotland.gov.uk/News/Releases/2013/03/benefit-cuts-24032013# (accessed 26 July 2013).

15 Northern Ireland Assembly Official Report, 25 November 2008.

16 This is a narrative in UK Coalition government policies. It is variously concerned with redefining the role of the market, voluntary sector and state and addressing anti-social behaviour (‘Broken Britain’), etc. For a discussion, see Smith 2010.

17 Excluding, for the present, income maintenance benefits or ‘social security’ (apart from limited competence in the case of Northern Ireland).
